# Clinical trial update on bispecific antibodies, antibody-drug conjugates, and antibody-containing regimens for acute lymphoblastic leukemia

**DOI:** 10.1186/s13045-019-0703-z

**Published:** 2019-02-08

**Authors:** Delong Liu, Juanjuan Zhao, Yongping Song, Xiaofeng Luo, Ting Yang

**Affiliations:** 1 Department of Oncology, The First Affiliated Hospital of Zhengzhou University, Zhengzhou, 450052 China; 2 Department of Hematology, The Affiliated Cancer Hospital of Zhengzhou University and Henan Cancer Hospital, Zhengzhou, China; 3 Department of Hematology, Fujian Institute of Hematology, Fujian Provincial Key Laboratory of Hematology, Fujian Medical University Union Hospital, Fuzhou, 350001 Fujian China

**Keywords:** Acute lymphoblastic leukemia, Bispecific antibody, Antibody-drug conjugate, Chimeric antigen receptor, Hyper-CVAD

## Abstract

The relapse rate remains high after chemotherapy for adult patients with acute lymphoblastic leukemia (ALL). With better molecular diagnosis and classification as well as better assessment for minimal residual disease, major progress in the treatment for refractory and/or relapsed ALL is being made. In addition to the tyrosine kinase inhibitors (TKIs) for Philadelphia chromosome-positive ALL, immunotherapeutic agents, blinatumomab, inotuzumab ozogamicin (INO), and chimeric antigen receptor (CAR) T cells, are changing the treatment paradigm for ALL. Blinatumomab and INO are being incorporated into induction chemotherapy regimens and combined with TKIs for ALL therapy. A novel low-intensity regimen, miniHCVD-INO-blinatumomab, appears to be less toxic and more effective than conventional intensive chemotherapy regimens. This review summarized new therapeutic researches of ALL and updated latest progress in clinical trials on bispecific antibodies, antibody-drug conjugates, and new regimens incorporating these novel antibodies.

## Background

Although high response rate is achieved from the chemotherapy for acute lymphoblastic leukemia (ALL), there is still high relapse rate for adult ALL patients [1–6]. The prognosis of adults with relapsed/refractory (R/R) ALL is still very poor [7, 8]. With better molecular diagnosis and classification as well as better assessment for minimal residual disease, major progress in the treatment for R/R ALL is being made [9–11]. Five tyrosine kinase inhibitors (TKIs) are available for chronic myeloid leukemia, and TKIs have made clear contribution to the improvement of outcome in patients with Philadelphia chromosome-positive (Ph+) ALL [12–18]. In addition to the TKIs, immunotherapeutic agents, blinatumomab, inotuzumab ozogamicin (INO), and chimeric antigen receptor (CAR) T cells are changing the treatment paradigm for ALL [19–29]. Hyper-CVAD is a commonly used chemotherapy regimen for ALL and has served as a backbone for the development of new regimens [30–36]. When CD20 expression is present, rituximab is added to chemotherapy regimens [37–41]. Blinatumomab and INO are being investigated in clinical trials for their incorporation into chemotherapy regimens and combined with TKIs for ALL therapy. The new low-intensity combination regimen, miniHCVD-INO-blinatumomab, appears to be less toxic and more effective. This review summarized new therapeutic researches of ALL and updated latest progress in clinical trials on bispecific antibodies, antibody-drug conjugates, and new regimens incorporating these novel antibodies.

### Bispecific T cell-engaging (BiTE) antibodies

#### Blinatumomab

The human CD19 antigen is a transmembrane protein expressed from pre-B cells until the terminal differentiation to plasma cells [42]. CD19 is a critical component of B cell receptor multicomplex [43]. Therefore, CD19 remains as the most reliable surface biomarker for B cells [44, 45]. CD19 is the most commonly targeted antigen to date in immunotherapy for hematological malignancies [46–48].

Blinatumomab (blina) is a novel first-in-human BiTE antibody against CD19/CD3 that is designed to bind specifically to CD19+ B cells and CD3+ T cells, resulting in T cell activation and a cytotoxic T cell response against CD19-expressing cells [49]. Blina is produced through recombinant DNA technology [50–54]. Blina is an antibody fragment with a molecular weight of 55 kDa. Therefore, its biological half-life is short and continuous IV infusion is required [55, 56]. Similar to the infusion of CAR T cells, cytokine release syndrome (CRS) and neurotoxicity are the major adverse events from blina therapy [25, 57–60]. In order to minimize CRS and neurotoxicities, it is recommended that the drug be used in a dose-escalating manner, with 9 μg/day for the first week, followed by 28 μg/day for the remaining 3 weeks [25, 51, 59]. Two weeks of treatment-free interval are recommended prior to the subsequent cycle. The FDA approved indications, dose, and schedules of blinatumomab administration are summarized in Table 1.

**Table 1:** Blinatumomab for B cell precursor acute lymphoblastic leukemia

	Weight ≥ 45 kg (fixed dose)	Weight < 45 kg (BSA-based dose)
CR1 or CR2 with *MRD-positive patients		
Cycles 1–4		
Days 1–28	28 μg/day	15 μg/m^2^/day (not to exceed 28 μg/day)
Days 29–42	14-day treatment-free interval	14-day treatment-free interval
Relapsed or refractory patients		
Induction cycle 1		
Days 1–7	9 μg/day	5 μg/m^2^/day (not to exceed 9 μg/day)
Days 8–28	28 μg/day	15 μg/m^2^/day (not to exceed 28 μg/day)
Days 29–42	14-day treatment-free interval	14-day treatment-free interval
Induction cycle 2		
Days 1–28	28 μg/day	15 μg/m^2^/day (not to exceed 28 μg/day)
Days 29–42	14-day treatment-free interval	14-day treatment-free interval
Consolidation cycles 3–5		
Days 1–28	28 μg/day	15 μg/m^2^/day (not to exceed 28 μg/day)
Days 29–42	14-day treatment-free interval	14-day treatment-free interval
Continued therapy cycles 6–9		
Days 1–28	28 μg/day	15 μg/m^2^/day (not to exceed 28 μg/day)
Days 29–84	56-day treatment-free interval	56-day treatment-free interval

*CR* complete remission, *MRD* minimal residual disease

*MRD is positive when blasts are ≥ 0.1% by flow cytometry in the bone marrow

A phase II multicenter clinical trial evaluating the safety and efficacy of blinatumomab in adult R/R Ph− B-ALL reported 43% CR rate [59]. Among these CR patients, 24–46% were then able to receive allogeneic hematopoietic stem cell transplantation (allo-HSCT) [25, 59, 61]. Blinatumomab is thus considered as an effective bridge therapy to allo-HSCT. The US FDA approved blinatumomab for the treatment of adult R/R Ph− B-ALL based on the phase II study [59]. Subsequently, a large randomized phase III trial comparing blinatumomab versus salvage chemotherapy for R/R B-ALL was reported [25]. This study enrolled 405 patients and randomized patients in a 2:1 ratio to receive blinatumomab (271 patients) or chemotherapy (134 patients). Compared to the chemotherapy group, the blinatumomab group had a significantly longer overall survival (OS) (7.7 months vs 4.0 months, HR 0.71, *p* = 0.01) and a higher complete remission (CR) rate (44% vs 25%, *p* < 0.001), further supporting blinatumomab as an efficacious and well-tolerated single-agent treatment option for R/R ALL.

Blina was also studied in B-ALL patients who had MRD+ after 3 months’ frontline therapy or those who relapsed with MRD disease [61]. These patients were treated with blina following standard protocols up to 4 additional cycles. Patients who did not receive allo-HSCT were given maintenance blina therapy every 3 months for 4 cycles (a total of 9 cycles). TKI was added for Ph+ ALL patients at the discretion of treating physicians. A total of 17 patients were enrolled, with 3 Ph+ patients. Thirteen out of 17 patients (76%) achieved MRD negativity, with 12 of the 13 patients being MRD negative after the first cycle. Six of the 13 (46%) patients received allo-HSCT due to donor availability. In a separate multicenter open-label single-arm study from Europe, blina treatment was given to adult B cell ALL patients with +MRD in CR1 or CR 2/3 which was defined by flow cytometry or PCR [62]. Among the 113 evaluable patients treated with blina, 88 patients (78%) achieved MRD negativity. The median OS was 36.5 months. When the patients with complete MRD negativity (MRD responders) were compared with those in persistent +MRD (non-responders), MRD responders had longer relapse-free survival (RFS) (23.6 vs 5.7 months; *p* = .002) and OS (38.9 vs 12.5 months; *p* = .002) than those in MRD non-responders. Therefore, it appears that blina is a good option to eliminate MRD after initial induction chemotherapy [63].

Blinatumomab has been approved for Ph− B cell R/R ALL. To evaluate the activity of blina in Ph+ R/R ALL patients, a phase II trial was started. The primary end point of the study was CR/CRh. In the first report of 45 such patients, CR/CRh was shown to be 36% (95% CI, 22% to 51%) during the first 2 cycles [64]. The responders included four of ten patients with the T315I mutation. Furthermore, the quality of the responses was striking, with MRD negativity in 88% of CR/CRh responders. Seven of the 16 responders (44%) proceeded to allo-HSCT. The secondary endpoints of the study included median RFS and OS, which were 6.7 and 7.1 months, respectively. The major adverse events were similar to those reported in Ph− R/R ALL patients. The CRS and neurotoxicities were mild without grade 4 or 5 neurologic events.

Nevertheless, the 43% CR rate from blina in R/R ALL means that a significant proportion of patients was still considered treatment-resistant. The mechanisms of resistance to blinatumomab therapy are still poorly understood. One case report described that blinatumomab treatment failure was associated with an increase in number of ALL cells positive for programmed death-ligand 1 (PD-L1), which may be one of the underlying immune escape mechanisms [65]. This indicates that further investigation on the therapeutic potential of inhibitors of immune checkpoint molecules needs to be considered to overcome blinatumomab resistance. Another mechanism may be due to CD19 antigen loss as this was reported in relapsed patients after blina therapy [66, 67], though this appears to be less common than those seen after CAR T therapy [28, 68, 69]. To overcome this problem, a number of ongoing studies are evaluating combination of blinatumomab with chemotherapy for Ph− ALL and with TKI for Ph+ ALL.

A recent update reported the outcome of blina treatment in pediatric patients with R/R B ALL in an expanded access study [70]. Treatment-emergent (TE) and treatment-related (TR) adverse events (AEs) were the primary endpoints. Morphologic CR and MRD response by PCR or flow cytometry were the secondary endpoints. At the time of the report, 98 patients were treated (median age, 8.5 [range 0.4–17.0] years). The median follow-up was 12.2 months (range 0.5–14.1). These patients were heavily pretreated. A median of 2 cycles (range 1–5) were administered, with 4 patients completing 5 cycles of blina. Virtually all patients experienced a TEAE whereas 77% had TRAEs. CRS was seen in 16%, with 2% severe. There were 9 grade 5 fatal AEs unrelated to blina treatment. Among the 98 patients, 60% (*n* = 59) achieved CR and 48% had MRD negativity. Among the 59 CR patients, 27 (46%) proceeded to allo-HSCT. The median OS was 13.0 months. In conclusion, blina induced MRD negativity in almost half of the patients, including patients with t(17;19). In this study, 4 patients who had prior blina treatment were re-treated and 3 achieved CR again. Blina has now been approved as a treatment option for pediatric patients with R/R ALL (Table 1).

Another retrospective analysis reported data on blina for CNS disease in 11 patients with B ALL [71]. Among the 11 patients, 10 had R/R ALL, 6 had Ph− ALL and 3 with Ph+ ALL. Of the 11 patients, 10 had systemic disease, only 1 with CNS only disease. Among the 11 patients who received blina, 3 had single agent blina, 4 in combination with BCR-ABL TKI, and 4 in combination with systemic chemotherapy. Intrathecal chemotherapy was given to all patients. Severe CNS toxicity was reported in 2 patients. Five of the 6 patients (83%) with active CNS disease became negative in CSF after blina therapy. There were 3 patients with positive CNS leukemic involvement on imaging studies. Among these 3 patients, 1 had CR, 1 had PR, and 1 had signs of inflammation. In conclusion, blina is safe and effective in B ALL patients with active CNS disease when given in combination with systemic and intrathecal chemotherapy. Currently, it remains unclear whether blina can penetrate the blood-brain barrier. In this regard, CAR T cells appear to have an advantage for CNS disease since it has been reported that CAR T cells were found in the CSF and are effective against CNS diseases in both hematological and solid tumors [72–75].

CRS is one of the major complications from blina therapy [50, 59]. IL-6 cytokine is found to be elevated and believed to be a major mediator of CRS [74, 76]. In a recent analysis updated at the 2018 ASH Annual Meeting, CRS was reported in 39 cases out of 1000 patients treated with blina [77]. Six of the 39 cases received tocilizumab, an IL-6 receptor antibody, 3 of the 6 patients received concurrent corticosteroids. Blina was interrupted and later restarted once CRS is resolved. CRS in all 6 cases resolved, and 3 cases resumed blina infusion. Among the 6 cases, 4 cases discontinued blina therapy. Recent studies on CAR T related toxicities have indicated that IL-1 and IL-6 are mediators of CRS, and IL-6 antagonist does not abrogate cytotoxicity of CAR T cells [57, 78, 79]. It is therefore possible that tocilizumab and IL-1 receptor antagonist may be used early as prophylaxis in addition to therapy. Clinical studies are ongoing for these clinical applications [57].

### Novel BiTE antibodies in pre-clinical development

#### CD3 X CD19 bi-valent BiTE

To enhance binding to leukemia cells expressing low-level CD19 molecules, bi-valent CD3 x CD19 BiTE antibodies, A-329, were produced [80]. The bi-valent A-329 BiTE was shown to be more potent in CD19 binding than the mono-valent format A-319 and in vitro human B cell killing (EC50 0.2 pM vs 3.4 pM). A-329 was confirmed to have greater cytotoxicity against a human diffuse large B cell lymphoma (DLBCL) cell line with a low expression of CD19 antigen. In monkey studies, the CD19 bi-valent A-329 BiTE was confirmed in vivo to have potent B cell killing, yet the adverse events were comparable between the mono-valent and bi-valent BiTE antibodies. In conclusion, the bi-valent A-329 appeared to have advantage in targeting tumor stem cells with low CD19 expression.

#### CD3 x CD20 full-length BiTE

DuoBody®-CD3xCD20 (GEN3013) is a novel BiTE targeting CD20-expressing B cells [81]. Different from blina, this DuoBody is a full-length bispecific IgG1 immunoglobulin with an effector function-silenced Fc region. This full-length BiTE was shown to be highly active in vitro with low picomolar EC_50_ towards a diverse panel of B cell lines.

#### CD3 x CD79b BiTE

CD79b is a pan-B cell marker and a component of the B cell receptor complex. CD79b is commonly used for the diagnosis of B cell leukemia and lymphomas. CD3 x CD79b BiTE was shown to induce T cell-dependent cytotoxicity towards CD79b expressing B cells [82]. The BiTE was shown to be active against B cell malignant cell lines as well as primary cells from B cell leukemia and lymphomas. These preclinical data were promising and further studies are needed for clinical applications.

### Antibody-drug conjugate (ADC) against CD22

CD22 is a common biomarker for B cells [83, 84]. It is expressed in B cells and in most cases of B-ALL. CD19 antigen loss has been observed to be a common mechanism of relapse after CD19-directed BiTE and CAR T cell therapies, yet CD22 remains detected in such cases. Therefore, CD22 antigen serves as a good target to treat R/R ALL [85].

Inotuzumab ozogamicin (INO) is an antibody-drug conjugate that consists of a humanized anti-CD22 monoclonal antibody linked to a cytotoxic agent calicheamicin which can cause double-strand DNA breaks and lead to apoptosis [86–88]. After the conjugate antibody binds to CD22, the CD22-conjugate complex is rapidly internalized. The calicheamicin is then released and results in apoptosis. In a large randomized phase III trial in adults with R/R ALL, single-agent INO was compared with commonly used salvage chemotherapy regimens [26]. The study demonstrated a significantly higher CR rate in the INO group than that in the chemotherapy group (80.7% vs 29.4%; *p* < 0.001), and a longer duration of remission (4.6 months vs 3.1 months; *p* = 0.03). INO has been approved to treat adult R/R pre-B-ALL (Table 2). Sino-occlusive syndrome (SOS, also known as veno-occlusive disease (VOD)) with liver function abnormality was reported to be a major adverse event [89, 90]. This treatment should be carefully planned particularly when allo-HSCT is being considered to minimize SOS complications. For patients planning to receive an allogeneic transplant, treatment with inotuzumab ozogamicin should be limited to 2 cycles of induction or the fewest number of cycles required to achieve a CR/CRi (if CR/CRi is not achieved after 2 cycles).

**Table 2:** Inotuzumab ozogamicin for relapsed or refractory B cell acute lymphoblastic leukemia

	Day 1	Day 8	Day 15
Induction*			
Cycle 1	0.8 mg/m^2^	0.5 mg/m^2^	0.5 mg/m^2^
Cycle length	21 days		
Consolidation^#^			
Dose	0.5 mg/m^2^	0.5 mg/m^2^	0.5 mg/m^2^
Cycle length	28 days		

*This may be repeated if patients do not achieve CR/CRi after cycle 1, though cycle length after cycle 1 should be 28 days

^#^For patients planning to receive an allogeneic transplant, treatment with inotuzumab ozogamicin should be limited to 2 cycles of induction or the fewest number of cycles required to achieve a CR/CRi (if CR/CRi not achieved after 2 cycles)

There are three immunotherapy options currently available for R/R B cell ALL, blina, INO, CD19-targeted CAR T, tisagenlecleucel [25, 26, 28, 68, 69]. It has been reported that tisagenlecleucel is effective in those R/R ALL patients who have failed blina [91]. “Off-the-shelf” third-party universal CAR T cells were also reported to be effective in R/R ALL who have failed blina therapy [92–94]. It remains unclear whether INO is effective in R/R ALL refractory to blina single agent. One case report described effective rescue with INO for a patient with R/R ALL from CML blast crisis who has failed blina therapy [95]. In this blina-refractory ALL, INO induced morphological CR but MRD remained positive.

### Novel ADC antibodies targeting B cells in preclinical and clinical development

Since CD79b is highly prevalent in B cell leukemia and lymphomas, CD79b-targeted ADCs have been in active preclinical and clinical development. The cytotoxic moieties include MMAE and DM1 that inhibit microtubule polymerization (polatuzumab vedotin, DCDS4501A) [96–99]. These CD79b-targeted ADCs have been tested in phase I/II clinical trials in B cell lymphomas. Early results are promising and the study on polatuzumab vedotin is completing (NCT01691898).

Another CD22 antibody was linked to MMAE in a new ADC targeting CD22 expressing B cells [98, 100]. The agent, pinatuzumab vedotin, was shown to be active in the preclinical studies. A multi-center, open-label, phase I study of this agent in B cell lymphoma and CLL has completed patient enrollment (NCT01209130).

### New regimens incorporating antibodies

#### Blinatumomab + TKI

Five TKIs have been approved for the treatment of chronic myeloid leukemia. These include imatinib, nilotinib, dasatinib, bosutinib, and ponatinib [101–103]. TKIs are routinely added to the therapy regimens for Ph+ ALL [1, 7, 18, 31]. Several studies are ongoing to evaluate the efficacy and safety of combination of TKIs (dasatinib or ponatinib) with blina for Ph+ ALL. These regimens may become chemo-sparing therapy for Ph+ ALL.

A retrospective analysis of French experience was recently reported in using blina + ponatinib (blina/pona) in 15 patients (9 male/6 female) with relapsed Ph+ ALL (2 blast crisis, 4 with T351I mutation) [104]. A median number of 3 cycles of blina was infused while ponatinib was given continuously. Among the 15 patients, 11 received an initial dose of 45 mg ponatinib once daily, 4 had 30 mg. A third of the patients stopped ponatinib and 47% stopped blina due to neurologic events. The cytogenetic CR was 93% (*n* = 14), and complete molecular CR was achieved in 12 patients. Two patients had CNS relapse while in molecular remission. The median follow-up for alive patients was 8 months (range 2.6–30.2) and the median OS was 8.5 months (range 1.7–30.2). In conclusion, the blina/pona combination was effective and tolerable in relapsed Ph+ ALL patients and may serve as an effective chemo-sparing salvage regimen. A prospective trial is ongoing (NCT03263572).

In a separate retrospective analysis of single center experience from the Memorial Sloan-Kettering Cancer Center, 11 patients (6 female/5 male) were identified. Among them, 7 received ponatinib, 3 dasatinib, and 1 nilotinib. Blina was infused at the standard dose and schedule [105]. Seven patients with +MRD turned MRD negative after blina/TKI therapy. Three patients proceeded to allo-HSCT. With a median follow-up of 7.7 months (range 3.2–16.0 months), the median OS has not been reached. Three patients developed grade 1 CRS and no neurotoxicity was observed. Relatively higher risk of transaminitis was seen in the blina/pona combination. In conclusion, this blina/TKI combination was safe and effective as a consolidation regimen for patients with MRD+ Ph+ ALL and may serve as a bridge therapy prior to allo-HSCT.

To further enhance the blina efficacy through immune checkpoint inhibition, a multi-center phase I dose-escalation study combining blina with nivolumab and ipilimumab is ongoing in R/R CD19+ ALL patients [106]. Patients with prior blina and/or allo-HSCT were also eligible. The treatment may continue up to 5 cycles of blinatumomab and 1 year of nivolumab/ipilimumab. Eight adults with a median age of 55 (range 25–75) were enrolled at dose level I. Transaminitis and chemical pancreatitis were observed in 20% of the patients. Infusion-related reactions to nivolumab were considered as a DLT. Among 5 evaluable patients, 4 achieved MRD negative CR. In conclusion, the blina/nivolumab combination in R/R ALL is safe and tolerable. Ipilimumab dose escalation is being started in this ongoing study.

### Antibodies + chemotherapy regimens

#### Rituximab + intensive chemotherapy

CD20 expression has served as a reliable biomarker for both diagnosis and therapy of B cell malignancies. In general, CD20 positivity is defined as expression of CD20 in greater than or equal to 20% of the blast cells in the NCCN guideline (www.NCCN.org). Rituximab is now routinely added to the chemotherapy regimens for CD20+ ALL and has been shown to increase event-free survival (EFS) [37, 38].

#### Blinatumomab + hyper-CVAD

To increase MRD negativity and decrease chemotherapy toxicities by reducing intensive chemotherapy, a phase 2 single-arm study was done to evaluate adding blina after completion of 4 cycles of hyper-CVAD for patients ≥ 14 years with newly diagnosed ALL [107]. After a total of 4 cycles of hyper-CVAD, 4 cycles of blina were administered as consolidation therapy. Intrathecal prophylaxis with methotrexate and cytarabine were given during the first 4 cycles of hyper-CVAD. In addition, rituximab or ofatumumab was added to hyper-CVAD cycles for those patients with CD20+ ALL (ofatumumab if CD20 ≥ 1% cells). For maintenance, POMP (6-mercaptopurine, vincristine, methotrexate, prednisone) was given on cycles 1–3, 5–7, 9–11, and 13–15 alternating with 3 cycles of blinatumomab on cycles 4, 8, and 12. The primary endpoint was RFS and secondary endpoints were OS, overall response rate (ORR), and MRD negativity rate.

At the last report, 17 patients were treated and only one patient had CD20 expression. Fourteen patients were evaluable. The ORR was 100% and the MRD negativity was 93% after the first cycle of therapy. A median of 4 cycles (range 1–4) of chemotherapy and 4 cycles (range 0–4) of blina were administered. No early death was reported. A total of 14 patients completed hyper-CVAD and initiated the blinatumomab phase. Among them, 9 patients have completed hyper-CVAD and blinatumomab sequential therapy and started maintenance therapy.

Two patients developed grade 3 adverse events attributable to blinatumomab. One patient had transient grade 3 CRS and the other had grade 3 ataxia. Both recovered after transient interruption of blinatumomab therapy and dexamethasone administration. The median follow-up was 14 months (range 3–20 months). At the update, OS was 94% among 17 patients and 14 of them in the first CR. The 1-year RFS rate was 77% (95% CI 42–93%) and the 1-year OS rate was 90% (95% CI 47–99%). In conclusion, the sequential combination of hyper-CVAD and blinatumomab in newly diagnosed B-ALL is safe. The preliminary results are encouraging. This study also incorporated 3 cycles of blina in the maintenance phase. This study is important since it assesses the activity of blina both in consolidation and in maintenance in the context of reducing hyper-CVAD chemotherapy cycles to 4 from conventional 8 cycles. The POMP maintenance cycles are also reduced from usually 3 years to 12 months.

#### MiniHCVD-INO

To increase clinical benefit in R/R ALL patients and minimize chemotoxicities, INO was added to a modified hyper-CVAD regimen, miniHCVD in a phase II trial [108]. In the mini-hyper-CVD (miniHCVD) trial, anthracycline was omitted and dexamethasone was given at half dose, whereas methotrexate dose was reduced 75% and cytarabine reduced to 0.5 g/m2 q12 h × 4 doses. The doses and schedules of the agents are summarized in Table 3. ORR and OS were the primary end points. A total of 59 patients were included in the analysis, with a median age of 35 years (range 18–87 years). The ORR was 78% (59% CR) and 1-year OS rate was 46%. The quality of the response is of particular interest as the responders had a MRD negativity rate of 82%. There were hyperbilirubinemia (14%; *n* = 8) and VOD/SOS (*n* = 9, 15%). To minimize the toxicity, INO dosage was lowered and ursodiol was added later in the trial protocol. With a median follow-up of 24 months, the median OS reached 11 months. Not surprisingly, earlier initiation of therapy with this regimen led to better outcome. The study reported 1-year OS rates for patients treated in salvage 1, salvage 2, and salvage 3 or beyond at 57%, 26%, and 39%, respectively (*p* = .03). The median OS of patients in the first salvage was 17 months which is very encouraging when compared with 9 months in historical series treated with INO or blina in the same institution.

**Table 3:** MiniHCVD-inotuzumab ozogamicin regimen for acute lymphoblastic leukemia

Treatment type	Schedule	Drug	Dose
Intensive phase	Cycles 1, 3, 5, 7	Cyclophosphamide	150 mg/m^2^ every 12 h, days 1–3
		Dexamethasone	20 mg/d, days 1–4 and 11–14
		Vincristine	2 mg flat dose, days 1 and 8
	Cycles 2, 4, 6, 8	Methotrexate	250 mg/m^2^, day 1
		Cytarabine	0.5 g/m^2^ every 12 h, days 2 and 3
	Cycle 1	Inotuzumab	1.3 mg/m^2^, day 3
	Cycles 2, 3, 4	Inotuzumab	1.0 mg/m^2^, day 3
	Cycles 1, 2, 3, 4	Rituximab*	375 mg/m^2^, days 1 and 8
Maintenance therapy	Months 1–12	Vincristine	2 mg/d every month
	Months 1–12	Prednisone	50 mg/d for 5 days every month
	Months 1–36	6-Mercaptopurine	50 mg PO twice daily
	Months 1–36	Methotrexate	10 mg/m^2^ PO weekly
Central nervous system prophylaxis	Cycles 1, 3	IT MTX-AraC	MTX 12 mg on day 2, AraC 100 mg day 8
	Cycles 2, 4	IT AraC-MTX	AraC 100 mg on day 5, MTX 12 mg day 8
Supportive care	Cycles 1–8	Pegfilgrastim	6 mg subcutaneously day 4
	Cycles 1–5	Ursodiol	300 mg, 3 times daily

Note: *Rituximab was administered in patients with CD20 expression of 20% or higher. It was noted that the schedules of rituximab and CNS prophylaxis have been modified in the latest publications and variations exist since the trials are still ongoing [108, 109]. For details on correct doses and schedules, those from original publications should be followed

Inotuzumab denotes inotuzumab ozogamicin; *IT* intrathecal, *MTX* methotrexate, *AraC* cytarabine

The outcome of this study was compared through a post hoc inverse probability of treatment weighing analysis with similar patients treated with single agent INO (*n* = 84). The ORRs and 1-year OS rates of miniHCVD + INO were significantly better than those treated with INO alone [108]. The treatment appeared to be a good bridging therapy to induce patients into remission followed by allo-HSCT since 44% of the patients were able to receive subsequent allo-HSCT.

This miniHCVD-INO regimen is being studied in a phase 2 trial at the MD Anderson Cancer Center (Houston, TX, USA) in newly diagnosed elderly (≥ 60) ALL patients [109]. Progression-free survival (PFS) was the primary endpoint. Fifty-two patients were evaluable, median age 68 years (64–72). The ORR was 98%. After a median follow-up of 29 months (13–48), a 2-year PFS was 59%. The 2- and 3- year OS were 66% and 56%, respectively. Median PFS was 35 months (95% CI 16.3 to not reached) and the median OS was not reached. Prolonged thrombocytopenia was the most frequent severe adverse event (81%). SOS/VOD was observed (8%). Treatment-related death rate was 12%.

In real-world database analysis, majority of older patients with ALL did not receive cytotoxic chemotherapy, mostly attributable to the possibility that these patients were thought to be unsuitable for intensive chemotherapy by the treating physicians [110–113]. In one analysis with 235 patients who were treated with cytotoxic chemotherapy, the median OS was 10.2 months (95% CI 8.3–12.7) [114]. In this trial in an older patient population treated with miniHCVD-INO, the median OS was not yet reached after a median follow-up of 29 months. In the GMALL study which enrolled 268 older patients with Ph− ALL, a subset of 43 patients who received a modified chemotherapy regimen with optimized CNS prophylaxis and consolidation therapy, the CR rate was 86%, and 5-year OS was 52% [115]. These results are similar to those obtained from the miniHCVD-INO frontline trial in older patients. Therefore, this low-intensity miniHCVD-INO combination regimen may open a new avenue for induction therapy of older patients with B-ALL. The efficacy of this regimen should be confirmed in a randomized phase 3 study.

#### MiniHCVD-INO-Blinatumomab

**Fig. 1:**
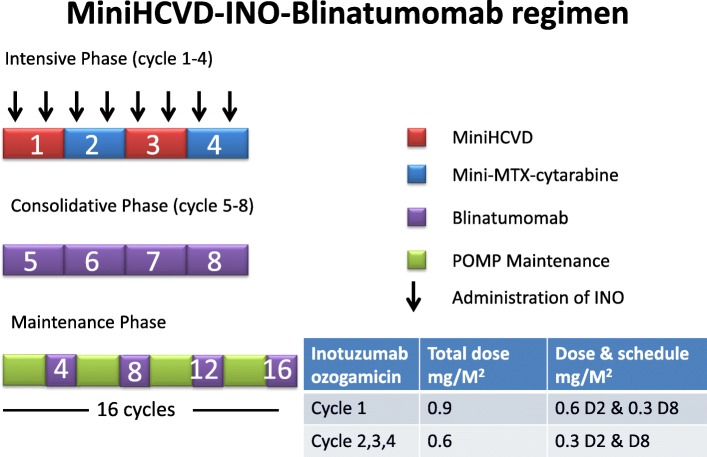
The diagrammatic schema of miniHCVD-inotuzumab ozogamicin-blinatumomab regimen. This was adapted from Jabbour et al. (2018) and Short et al. (2018). Detailed dosages and schedules are summarized in Table 4. miniHCVD low-dose hyper-fractionated cyclophosphamide, vincristine, dexamethasone. MTX methotrexate. INO inotuzumab ozogamicin; POMP prednisone, vincristine, methotrexate, mercaptopurine; D day

As a single agent, both INO and blina were shown in randomized studies to be better than salvage chemotherapies [25, 26]. Therefore, new trials are incorporating blinatumomab into the miniHCVD-INO regimen in an attempt to further improve the outcome for ALL patients [116–119]. In these studies, the treatment was divided into 3 phases: intensification, consolidation, and maintenance (Fig. 1, Table 4). Four cycles of intensive chemotherapy with miniHCVD as described above are given. Instead of monthly dosing, the INO dose is divided into day 2 and day 8 of each cycle (Fig. 1). The total dosage of INO was also reduced for each cycle. Blinatumomab is given in the 4 consolidation cycles, followed by maintenance with 4 more cycles given on month 4, 8, 12, and 16 (Table 4). The POMP-blina chemotherapy maintenance is given for a total of 16 cycles (Fig. 1, Table 4). Addition of blinatumomab makes it possible to reduce POMP maintenance cycles from 3 years to 12 months.

**Table 4:** miniHCVD-inotuzumab ozogamicin-blinatumomab regimen for acute lymphoblastic leukemia

Treatment type	Schedule	Drug	Dose
Intensive phase	Cycles 1, 3	Cyclophosphamide	150 mg/m^2^ every 12 h, days 1–3
		Dexamethasone	20 mg/d, days 1–4 and 11–14
		Vincristine	2 mg flat dose, days 1 and 8
	Cycles 2, 4	Methotrexate	250 mg/m^2^, day 1
		Cytarabine	0.5 g/m^2^ every 12 hours, days 2 and 3
	Cycle 1	Inotuzumab	0.6 mg/m^2^, day 2 & 0.3 mg/m^2^, day 8
	Cycles 2, 3, 4	Inotuzumab	0.3 mg/m^2^, days 2 and 8
	Cycles 1, 2, 3, 4	Rituximab*	375 mg /m^2^ day 1 and 8
Consolidation phase	Cycle 5	Blinatumomab	9 μg/day, days 1–4; 28 μg/day days 5–28
	Cycles 6, 7, 8	Blinatumomab	28 μg/day days 1–28
Maintenance therapy	Months 1–3, 5–7, 9–11, 13–15	Vincristine	2 mg/d every month
	Months 1–3, 5–7, 9–11, 13–15	Prednisone	50 mg/d for 5 days every month
	Months 4, 8, 12, 16	Blinatumomab	28 μg/day days 1–28
	Months 1–3, 5–7, 9–11, 13–15	6-Mercaptopurine	50 mg PO twice daily
	Months 1–3, 5–7, 9–11, 13–15	Methotrexate	10 mg/m^2^ PO weekly
Central nervous system prophylaxis	Cycles 1, 3	IT MTX-AraC	MTX 12 mg on day 2, AraC 100 mg day 8
	Cycles 2, 4	IT AraC-MTX	AraC 100 mg on day 5, MTX 12 mg day 8
Supportive care	Cycles 1–4	Pegfilgrastim	6 mg subcutaneously day 4
	Cycles 1–5	Ursodiol	300 mg, 3 times daily

Note: For Intensive phase, inotuzumab ozogamicin (INO) is administered in two split doses on day 2 and day 8. The first dose of INO is 0.6 mg/m2, each subsequent dose is 0.3 mg /m2. In the Consolidation phase, blinatumomab was initiated at 9 μg/day for 4 days and then escalated to 28 μg/day by continuous IV infusion for a total of 4 weeks followed by 2 weeks treatment-free interval, and the cycle is repeated every 6 weeks. *Rituximab was administered in patients with CD20 expression of 20% or higher. It was noted that the schedules of rituximab and CNS prophylaxis have been modified in the latest publications, and variations exist since the trials are still ongoing [116, 117]. For details on correct doses and schedules, those from original publications should be followed

Inotuzumab denotes inotuzumab ozogamicin; *IT* intrathecal, *MTX* methotrexate, *AraC* cytarabine

In the combination trial of miniHCVD + INO + blina for newly diagnosed older ALL patients, 58 patients were treated at the last report [117]. Among the 58 patients, 31 had CD20 expression ≥ 20% and received rituximab. Fifty-four patients were evaluable for morphological responses. The ORR was 95% (*n* = 53, CR, *n* = 47; CRp, n = 5; CRi, *n* = 1). The overall MRD negativity was 95% in 57 evaluable patients. There was no day-30 mortality. Among the 57 patients with CR, 8 relapsed, 3 proceeded to allo-HSCT, and 31 continued on therapy or completed maintenance. A total of 17 patients died in CR/CRp. The rate of SOS was 8–11%. The median follow-up was 28 months (2–68 months). The 3-year OS rate was 54%. When this result was compared to a similar historical cohort of older patients treated with hyper-CVAD ± rituximab (*n* = 77), the miniHCVD + INO ± blina led to significantly higher 3-year OS (54% vs 32%; *p* = 0.002). This new combination regimen appears to be safe and effective in elderly patients with newly diagnosed Ph− ALL. Randomized studies are needed to confirm this new immunotherapy-based lighter chemotherapy.

The miniHCVD + INO ± blina regimen is ongoing in R/R ALL and has been recently updated [116, 118]. A total of 84 patients were treated including 17 patients with miniHCVD + INO + blina [118]. The treatment schedule and dosages have been published and are summarized in Fig. 1 and Table 4 [116]. The median age was 35 (range 9–87), and the median follow-up was 31 months (range 0.1–64.1). These patients were heavily pretreated and 23% of them had failed prior allo-HSCT. The ORR was 80% (CR, 58%; CRp/CRi, 21%), and 81% achieved MRD negativity, with better response in earlier lines of salvage therapy. Thirty-four patients (40%) proceeded to allo-HSCT. Three-year OS was 33%. SOS rate was reduced from 15% to 0% when the INO dose was split to two doses each cycle. This study showed again that this low-intensity immunotherapy-containing miniHCVD + INO + blina is safe and effective in R/R heavily pretreated ALL patients. In addition, 4 cycles of blinatumomab as consolidation therapy increase the interval between the last dose of inotuzumab ozogamicin and allo-HSCT. The long interval between INO and allo-HSCT as well as split-dose INO appears to markedly reduce SOS risk. More patients are needed for the triple combination regimen to better assess the risk of adding the antibodies, and randomized study is need to ascertain the value of this novel combination regimen.

When the patients who were treated as first salvage on this regimen were analyzed (*n* = 48), ORR was 92% and CR 73% [116]. MRD negativity was 93%. With a median follow-up of 31 months, the median OS was 25 months. Half of the 48 patients proceeded to allo-HSCT. This outcome was compared with historical controls of similar patients treated in the same institution with either salvage chemotherapy or INO, and the combination regimen miniHCVD-INO +/− blina had better outcome than the intensive salvage chemotherapy or INO alone. Therefore, this low-intensity regimen appears to lead to better outcome for first salvage therapy for R/R ALL patients. The results however require confirmation in randomized studies.

In summary, it appears that this low-intensity chemotherapy regimen with 4 cycles of miniHCVD + mini-methotrexate-cytarabine in combination with immunotherapy is promising. The trials are ongoing and the regimen is still going through modifications. The modifications included splitting INO dosage to minimize SOS/VOD, and blinatumomab was added to consolidation phase as well as to the maintenance phase for better elimination of MRD. CD20 expression is typically considered positive in 20% cells and rituximab was added in those cases [108, 116]. It appears that the schedules of rituximab and CNS prophylaxis had variations in miniHCVD-INO-blina low-intensity regimen when compared with those used in conventional hyper-CVAD regimens.

## Conclusions and future perspectives

TKIs, bispecific antibodies, antibody-drug conjugates, and CAR T cells are changing the treatment paradigm for ALL. More targeted agents including BTK inhibitors (ibrutinib, acalabrutinib), BCL-2 inhibitor (venetoclax), and immune checkpoint inhibitors are being studied for ALL therapy [120–123]. It remains unclear at this time how to position and sequence these new agents and regimens. When and how to use allo-HSCT in the overall treatment algorithm of B cell precursor ALL is another challenge. The current trend points to the direction to use less cytotoxic chemotherapy and more targeted agents as well as immunotherapeutic agents including blina, INO, and CAR T cells. Studies are ongoing to use these agents in frontline settings, particularly in older patient population. It is foreseeable that with targeted, more efficacious and less toxic regimens, the outcome from ALL therapy will be significantly improved.

## Abbreviations

ADC: Antibody-drug conjugate
ALL: Acute lymphoblastic leukemia
CAR: Chimeric antigen receptor
CRS: Cytokine release syndrome
